# An Integrated Physics-Based and Data-Driven Framework for Defect Prediction in Advanced Nanoimprint Lithography Toward Inorganic Semiconductor Patterning

**DOI:** 10.3390/mi17060674

**Published:** 2026-05-29

**Authors:** Jean Chien, Eric Lee

**Affiliations:** Department of Chemical Engineering, National Taiwan University, Taipei 10617, Taiwan; f12524060@ntu.edu.tw

**Keywords:** nanoimprint lithography (NIL), electron-beam lithography (EBL), reactive ion etching (RIE), physics-augmented convolutional neural network, virtual inspection, defect prediction, physics-based process twin, uncertainty calibration

## Abstract

Advanced nanoimprint lithography (NIL) is promising for inorganic semiconductor patterning because it enables high-resolution replication with a relatively simple process flow; however, yield loss increasingly originates from spatially distributed, subcritical distortions accumulated across coating, exposure, etching, and imprinting. In this study, we propose an integrated physics-based and data-driven framework for pre-manufacturing defect-risk prediction in NIL. The framework combines an NDA-safe layout database, a physics-based process twin, and a stochastic risk prediction model using a physics-augmented convolutional neural network with conformal uncertainty calibration. Starting from binary design layouts, the process twin sequentially captures resist thickness variations during spin coating, proximity-induced dose redistribution and development-induced pattern deformation during electron-beam lithography (EBL), density-sensitive pattern transfer during reactive ion etching (RIE), and three-dimensional resist filling during imprinting, thereby generating physically consistent parameter maps for downstream learning. The results demonstrate an end-to-end virtual inspection flow that converts layouts into spatially resolved risk maps before fabrication. In addition, patterns with similar contour extent but different local density exhibit distinctly different risk distributions, indicating that manufacturability is governed not only by nominal geometry but also by local pattern environment. These findings support pre-manufacturing virtual inspection as a physically interpretable route for early yield-risk screening in advanced NIL.

## 1. Introduction

NIL has emerged as a promising patterning route for advanced semiconductor manufacturing because it provides high-resolution replication with a comparatively simple process architecture. In contrast to projection-based optical lithography, NIL transfers structural information through direct mold-defined patterning, which makes it especially attractive for dense and repetitive geometries such as line/space arrays, contact arrays, and other memory-oriented layouts. This advantage becomes increasingly relevant for inorganic semiconductor patterning, where nanoscale geometric fidelity must be maintained over large pattern populations and across tightly coupled process steps. At the same time, the corresponding yield risk is no longer dominated only by catastrophic failures. Instead, it often originates from spatially distributed, subcritical distortions that accumulate across coating, exposure, etching, and imprinting before they become functionally significant [1,2,3,4,5,6,7,8,9,10].

Conventional inspection strategies remain predominantly post hoc. In most industrial settings, defects are identified only after fabrication through SEM-based review, optical inspection, or subsequent defect classification pipelines. Although these approaches are effective for post-process monitoring, they inherently limit early intervention because wafers have already passed through multiple costly process steps by the time the defect signal becomes observable. Beyond the mere binary of failure, the gradual nature of process-induced degradation, as manifested in critical-dimension (CD) drift and line-edge distortion, remains largely obscured by conventional metrics. This oversight is amplified in the context of advanced NIL, where high-fidelity requirements dictate that even a singular, subcritical anomaly on a template can propagate into a systemic yield loss [11,12,13].

Recent studies have approached this challenge from two partially separate directions. On one side, physics-based simulation has been used to analyze individual process steps, including resist coating, lithographic proximity effects, etch microloading, and resist flow during imprinting [1,14,15,16,17]. Such models provide mechanistic insight into how local geometry interacts with material transport and process conditions, where finite-element and multiphase-flow frameworks quantify the influence of viscosity, pressure distribution, and feature geometry on filling dynamics and defect formation [18,19], while structured-liquid and level-set-based approaches further enable time-dependent tracking of interface evolution and viscoelastic deformation during imprint processes [20]. At smaller scales, molecular dynamics simulations reveal how local polymer composition and viscosity govern nanoscale filling kinetics and structural homogeneity in confined geometries [21], and mechanical analyses of stamp deformation highlight how tool-level non-uniformity contributes to residual-layer variation and pattern fidelity degradation [22].

On the other side, data-driven methods have been developed for layout-aware inspection enhancement, virtual metrology, and layout-to-SEM image reconstruction, especially when uncertainty quantification (UQ) is incorporated into the learning framework [2,23,24,25]. Deep learning models, including CNNs and U-Net-based architectures, have demonstrated the capability to predict SEM-like images directly from layout inputs, capturing layout-dependent features such as line-edge roughness and proximity-induced distortions [26]. More recent studies introduce uncertainty-aware formulations, including quantile-based prediction and Bayesian inference, to provide pixel-level confidence estimation for virtual metrology tasks [27,28], while generative models such as GAN-based frameworks further enhance the realism and diversity of synthesized SEM images under limited training data conditions [29]. In addition, OPC-aware layout-to-SEM prediction approaches incorporate process-corrected geometries to improve consistency between predicted and measured pattern responses under varying lithographic conditions [30].

Beyond reconstruction, process-aware defect-prediction methods have been developed to directly identify failure-prone regions prior to fabrication, leveraging layout topology, pattern density, and machine learning techniques. Graph-based models encode spatial relationships between pattern features to predict layout-dependent failure modes such as bridging and necking [31,32], while OPC-aware hotspot-detection frameworks use layout embeddings to capture non-local interactions between pattern geometries and process variations [33]. Density-dependent models further demonstrate that the local pattern environment strongly influences defect formation through mechanisms such as loading effects, transport limitation, and stress concentration [34]. Physics-augmented machine learning approaches integrate simplified process models with data-driven correction to improve prediction robustness under varying process conditions [35,36]. These studies collectively indicate that defect formation is not solely determined by nominal geometry, but is strongly modulated by local pattern context and cross-stage process interactions.

However, despite these advances, existing studies are largely limited to either stage-specific physical simulations or method-specific data-driven models, with limited integration across the full process chain. In particular, most physics-based models focus on individual steps such as imprint filling or etch transfer, whereas data-driven approaches primarily operate on layout–image mappings without explicit incorporation of upstream physical field evolution. As a result, the coupling between sequential processes—such as thickness variation affecting exposure, exposure affecting etch transfer, and etch topography influencing imprint behavior—remains insufficiently captured. Consequently, few frameworks explicitly connect layout-dependent physical-field evolution—from coating and exposure to etching and imprinting—to uncertainty-aware, spatially resolved defect-risk prediction. As a result, the ability to translate a design layout into a physically grounded, pre-fabrication risk map remains largely unexplored. To address this limitation and bridge the gap between stage-resolved physical modeling and data-driven risk inference, an integrated framework is required.

The present study addresses this gap by proposing an integrated physics-based and data-driven framework for defect prediction in advanced NIL toward inorganic semiconductor patterning. The framework combines three tightly coupled modules: an NDA-safe layout database, a physics-based process twin with parameterized physical models, and a stochastic risk prediction stage based on physics-informed CNN reconstruction with uncertainty calibration. The process twin preserves stage-resolved physical fields rather than reducing each process to a single scalar parameter, thereby retaining where and why pattern deformation develops. The final output is not merely a reconstructed SEM-like image, but a pixel-wise risk map that reflects both predicted distortion and calibrated uncertainty. This design reformulates defect prediction from a post hoc binary classification problem into a pre-manufacturing risk inference problem.

Two questions motivate the present work. First, can a design layout be translated into an end-to-end, physics-grounded virtual inspection result before wafers are processed? Second, even with similar contour extent, does the local pattern density still alter the eventual defect risk? As shown below, the answer to both questions is affirmative. The results demonstrate that physically interpretable, stage-resolved precursor maps can be combined with uncertainty-aware learning to generate actionable risk distributions before fabrication begins. They also show that patterns with similar contour can exhibit different vulnerability when the surrounding pattern environment changes, indicating that manufacturability is governed not only by apparent geometry but also by local process coupling.

## 2. Materials and Methods

### 2.1. Overall Framework

The proposed framework consists of three tightly connected components: an NDA-safe layout database generated by a physics-aware pattern synthesis engine, a physics-based process twin that propagates each layout through spin coating, EBL exposure/development, RIE transfer, and NIL deformation/curing, and a stochastic risk prediction model combining CNN-based virtual SEM reconstruction with uncertainty calibration. The overall objective is to map a layout not to a binary defect label, but to a spatially resolved defect-risk field supported by both physical precursors and calibrated prediction intervals.

**Figure 1:**
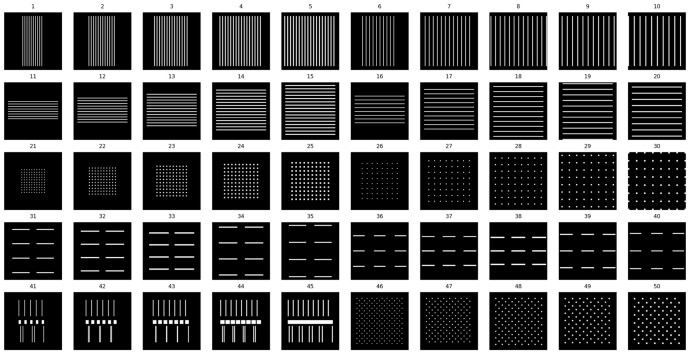
Representative NDA-safe memory layouts spanning dense- and relaxed-pitch line/space, contact, staggered contact, jog, gate-cut, and locally modulated density pattern families.

The NDA-safe pattern library contains 50 representative layout patterns, including isolated and dense line/space arrays, dense and relaxed contact arrays, staggered contacts, jog-like distortions, gate-cut-like motifs, and locally modulated density structures, as shown in Figure 1. To enhance data diversity while preserving physical consistency, each representative pattern is further augmented through rotational transformations (90°, 180°, and 270°) and scaling operations, resulting in an expanded dataset of 250 layout samples. All patterns are represented as 600 × 600 binary matrices, which allows direct transfer into the physics-based simulation chain without exposing proprietary mask data. After excluding reserved samples not used in model development, a total of 195 samples are used for CNN training and validation, while 30 samples are allocated for conformal calibration and 25 samples for testing. Specifically, the dataset is partitioned into 150 training samples, 45 validation samples, 30 calibration samples, and 25 testing samples. This dataset design ensures sufficient diversity for model learning, while maintaining a statistically consistent calibration set for uncertainty quantification. This is important because the study aims to preserve manufacturing relevance while remaining scalable and reproducible.

A key design principle of the framework is that each process stage outputs a spatial field rather than a single process indicator. The same nominal recipe is applied to all patterns, yet different layout environments generate different local responses. Therefore, the quantities passed to downstream stages are not just averaged parameters but pattern-conditioned maps. This choice is essential because the targeted end result is a spatial risk map; a scalar-only description would destroy the local information needed to explain or predict defect emergence.

### 2.2. Physics-Based Process Twin with Parameterized Physical Models

**Figure 2:**
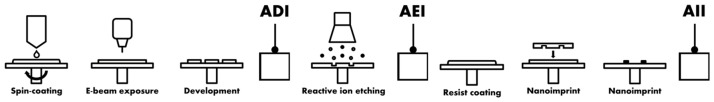
Integrated NIL workflow with ADI (After Development Inspection), AEI (After Etching Inspection), and AII (After Imprint Inspection).

Each NDA-safe layout is propagated through an integrated physics-based process twin that explicitly resolves the spatial fields responsible for pattern deformation across the NIL workflow. Rather than assigning a single scalar dominant parameter to each stage, the same physical recipe is applied to all patterns, while the layout geometry induces non-uniform process responses. Therefore, each stage yields a pattern-conditioned parameter map that serves as a process-sensitive descriptor for downstream variability modeling. In this sense, the process twin is not a collection of unrelated simulators. It is a sequential cascade in which thickness non-uniformity perturbs exposure response, exposure response perturbs the developed contour, the developed contour perturbs etch transfer, and the etched topography perturbs mold-driven resist filling during NIL. This stage-to-stage propagation structure provides the physical backbone of the proposed framework, as shown in Figure 2.

#### 2.2.1. Spin Coating: Resist Thickness Field as the First Process-Conditioned Prior

The first stage describes the coated resist as a thin liquid film undergoing centrifugal spreading, viscous dissipation, solvent evaporation, and edge-bead formation. Following the thin-film viewpoint used in the master-template simulation study, the local thickness h(r,t)  evolves from the balance between outward centrifugal driving and viscous resistance. Under the lubrication approximation, the depth-averaged radial flux can be written as

(1) $$q_{r}=\int_{0}^{h}u_{r}dz=\frac{h^{3}}{3\mu }\left(\rho \omega^{2}r-\partial_{r}p\right)$$

where *μ* is viscosity, *ρ* is density, *ω* is the spin speed, and *p* is pressure, with the driving terms corresponding to centrifugal force ($\rho \omega^{2}r$) and capillary pressure gradient ($\partial_{r}p$). Mass conservation then gives

(2) $$\frac{\partial h}{\partial t}+\frac{1}{r}\frac{\partial }{\partial r}\left(rq_{r}\right)=-E$$

where *E* denotes solvent evaporation. In the wafer interior, where capillary pressure gradients are weak ($\partial_{r}p\approx 0$), this reduces to the classical Emslie–Meyerhofer-type thinning form

(3) $$\frac{\partial h}{\partial t}=-\frac{2\rho \omega^{2}}{3\mu }h^{3}-E$$

This formulation offers a mechanistic window into the physical origin of the average thickness dependence on spin speed. Furthermore, it underscores the inherent inadequacy of treating spin coating as a single nominal thickness, revealing how the interplay between center-to-edge profiles and edge-bead dynamics dictates the downstream exposure threshold.

To preserve both global and local variation, the thickness profile is represented as the sum of a core thickness term and an edge correction:

(4) $$h\left(r,\omega \right)={\;h}_{c}\left(\omega \right)\left[1+a_{2}{\left(\frac{r}{R}\right)}^{2}+a_{4}{\left(\frac{r}{R}\right)}^{4}\right]+{\;h}_{e}\left(\omega \right)exp\left[-\frac{{\left(R-r\right)}^{2}}{2w_{e}{\left(\omega \right)}^{2}}\right]$$

Here,${\;h}_{c}\left(\omega \right)\;$ follows a power law consistent with Emslie–Meyerhofer scaling, while ${\;h}_{e}\left(\omega \right)\;$ and ${\;w}_{e}\left(\omega \right)\;$ represent the spin-speed-dependent amplitude and width of the edge bead. The model has been previously validated [2], and the associated parameters used in this study are summarized in Appendix A.1. The local 600 nm × 600 nm tile extracted from the wafer is then normalized as

(5) $$\hat{h}\left(x,y\right)=\frac{\left(h\left(x,y\right)-\bar{h}\right)}{\bar{t}}$$

which serves as the first pattern-conditioned process prior. Physically, this map reflects where the resist is locally thicker or thinner than the tile average, and therefore where dose-to-clear (i.e., the minimum exposure dose required to fully remove the resist during development) and subsequent filling dynamics may deviate from the nominal response.

A practical merit of this stage is that it establishes a unified interface variable for downstream coupling. Rather than manually adjusting later parameters by ad hoc bias, the framework carries forward the thickness deviation map explicitly. This improves consistency with the process chain and makes it possible to trace a later defect tendency back to an upstream coating perturbation.

#### 2.2.2. E-Beam Exposure and Development: Proximity-Driven Dose Redistribution Coupled to Thickness-Modulated Development

In the second stage, the coated layout is exposed by electron-beam lithography. The relevant physical mechanism is not merely nominal dose assignment, but spatial dose redistribution caused by forward scattering in the resist and backscattering in the substrate. The process twin therefore outputs both the deposited dose field D(x,y)  and the proximity-affected dose field $D_{prox}(x,y)$. This distinction matters because the local pattern response is governed by the dose that actually accumulates after scattering, not by the ideal shot map alone.

Following the integrated EBL treatment described in the master-template study, the effective dose distribution after proximity effects can be written in convolution form as

(6) $$D_{prox}(x,y)=D(x,y)*K_{PE}(x,y)$$

where $K_{PE}\;$ is a proximity kernel representing the combined effect of short-range forward scattering and long-range backscattering. The exact kernel form is modeled using the area-normalized hybrid proximity kernel adopted in the master-template simulation, which combines a Gaussian short-range component and a Lorentzian long-range component to represent forward scattering in the resist and backscattering in the substrate. As a result, the exposure field becomes spatially redistributed in a manner that depends on local pattern density and neighborhood geometry. Further details of the formulation are provided in our previous work [2]. Dense features therefore accumulate interaction dose more strongly than isolated ones, even under the same nominal recipe.

The coupling from spin coating enters through the development threshold. Instead of using a constant threshold, the local threshold is written as

(7) $$T\left(x,y\right)=T_{0}\left[1+\alpha \hat{h}(x,y)\right]$$

where $T_{0}$ is the nominal dose-to-clear threshold corresponding to the exposure dose distribution, and α is a coupling coefficient. The associated parameters used in this study are summarized in Appendix A.2. This relation expresses a simple but important physical point: the development outcome depends on both exposure and local resist condition. A thicker or thinner coated region changes the effective response, so development is represented as the competition between a proximity-redistributed dose field and a thickness-modulated threshold field. The ADI contour is then obtained by thresholding $D_{prox}(x,y)$ against T(x,y).

This stage is where CD bias, line thinning, and local edge distortion first emerge in a pattern-specific manner. Importantly, the framework retains the full dose fields as interpretable process maps. Therefore, when the final defect risk becomes high in a certain region, the model can distinguish whether the cause is associated with proximity accumulation, threshold sensitivity, or later transfer stages. That interpretability is one of the advantages of keeping D and $D_{prox}(x,y)$ as explicit priors rather than collapsing them into a single post-development binary image.

#### 2.2.3. Reactive Ion Etching: Density-Sensitive Pattern Transfer and Shrinkage Evolution

The third stage models the transfer from ADI to AEI using a level-set-based RIE module. The purpose of this stage is to capture geometry-dependent linewidth loss under anisotropic etching, shadowing, microloading, and aspect-ratio-dependent etching (ARDE). In the integrated master-template study, the RIE stage was described as a level-set evolution process whose outputs include CD shrinkage and transfer fidelity metrics. The results showed stronger shrinkage in dense patterns than in isolated ones under identical process conditions.

A standard interface-tracking description is to represent the evolving material surface using a level set function ϕ(x,y,z,t), where the zero-level set ϕ=0 defines the interface. The evolution of the interface follows a Hamilton–Jacobi-type equation:

(8) $$\frac{\partial \phi }{\partial t}+R_{etch}\left|\nabla \phi \right|=0$$

where $R_{etch}$ is the local etch rate at the interface and |∇ϕ| ensures propagation along the surface normal direction. The etch rate encodes the key physics of the RIE process, including surface orientation dependence, angular ion flux distribution, transport accessibility, and passivation effects.

The etch rate can be expressed in a compact form that captures anisotropic ion-driven etching and transport-limited effects as:

(9) $$R_{etch}=R_{base}\left[f_{ang}\left(\left|n_{z}\right|\right)f_{acc}\left(AR\right)\left|n_{z}\right|+r_{lat}f_{lat}\left(AR\right)\left(n_{x}^{2}+n_{y}^{2}\right)+f_{chem}\right]$$

where $\;f_{ang}$ represents the angular anisotropy of the ion flux, $f_{acc}\left(AR\right)$ captures ARDE-related transport limitation, and $f_{lat}\left(AR\right)$ accounts for lateral/passivation modulation. The associated parameters used in this study are summarized in Appendix A.3. The unit normal vector $\overset{⃑}{n}=(n_{x},\;n_{y},n_{z})$ is computed from the normalized gradient of the level set function.

Although the detailed parametrization is recipe-dependent, this structure captures the essential physical trend: as local pattern density increases, transport into narrow features becomes less efficient, leading to enhanced linewidth retreat and modified sidewall evolution.

The spatially resolved descriptor passed to downstream stages is defined as the CD shrinkage map:

(10) $$∆CD\left(x,y\right)={CD}_{AEI}\left(x,y\right)-{CD}_{ADI}\left(x,y\right)$$

where ${CD}_{ADI}\left(x,y\right)\;$ and ${CD}_{AEI}\left(x,y\right)\;$ denote the local critical dimension, defined here as the effective linewidth of the patterned feature at spatial position (x,y), after development inspection (ADI) and after etching inspection (AEI), respectively. A negative ∆CD(x,y)  therefore indicates linewidth reduction during the RIE transfer step. For a fixed analysis window, the same transfer loss can also be expressed using an area-based shrinkage ratio,

(11) $$S\equiv 1-\frac{A_{AEI}}{A_{ADI}}$$

where $A_{ADI}\;$ and $A_{AEI}\;$ represent the patterned area before and after etching within the same analysis window, respectively. Together, ∆CD(x,y)  and S summarize not only local linewidth reduction but also density-driven transfer degradation. The resulting AEI patterns typically exhibit narrower linewidths, stronger bilateral retreat, more pronounced line-end pullback, and increased gap opening in dense pattern environments.

In other words, the RIE stage is not merely a geometric transfer step. It is the first stage where the framework demonstrates that patterns with comparable geometric extent do not necessarily exhibit comparable transfer fidelity. The local pattern environment plays a critical role. By propagating ∆CD(x,y) into the downstream NIL and learning stages, the framework preserves density-dependent process variation rather than collapsing it into a final binary contour.

#### 2.2.4. Nanoimprint Lithography: Viscoelastic Filling, Interface Evolution, and Curing-Induced Morphology Locking

The fourth stage extends the integrated process chain into NIL itself. This is a crucial addition because the mold inherited from AEI does not simply replicate the final wafer structure without modification. Instead, the final morphology is governed by resist flow, interface transport, and curing-induced stabilization under mold constraints. In this study, the NIL stage is therefore modeled as a three-dimensional viscoelastic filling problem coupled to a level-set description of the resist–air interface. The main stage outputs are the evolving interface field ϕ(x,y,z,t) and the final resist height map H(x,y), which encode filling completeness, residual-layer non-uniformity, and void-prone regions.

The resist and air are separated by the sign of the level-set field:

(12) ϕ(x,y,z,t)<0 in the resist

(13) ϕ(x,y,z,t)>0 in air

(14) ϕ=0 at the interface

Before imprint, the resist is initialized as a flat film of thickness $\;h_{0}$. In production NIL (e.g., Canon J-FIL or SSIL), the imprint resist is not formed by spin coating but is dispensed as discrete droplets of low-viscosity UV-curable material [37]. If ∆z is the grid spacing in the vertical direction, and the initial number of filled layers is

(15) $$n_{f}=\left[\frac{h_{0}}{∆z}\right]$$

and the initial phase field is assigned such that the first $n_{f}\;$ layers correspond to the resist region, while the remaining domain represents air. This definition provides a consistent interface representation and allows the model to evolve, filling, trapping, and local geometry closure without explicit boundary tracking.

To represent the polymeric nature of the imprint resist, the model introduces an extra stress tensor τ and adopts a simplified Maxwell/Oldroyd-B-type relaxation form:

(16) $$\frac{\partial \tau }{\partial t}=\frac{2\eta \left(\phi \right)D-\tau }{\lambda }$$

where λ is the relaxation time, η(ϕ) is the phase-dependent effective viscosity, and

(17) $$D=\frac{1}{2}\left(\nabla \nu +{\left(\nabla \nu \right)}^{T}\right)$$

is the rate-of-deformation tensor defined from the velocity field $\nu =\left(v_{x},v_{y},v_{z}\right)$. This simplified form retains the two mechanisms most relevant to NIL filling at the present scale: local stress generation by deformation and gradual stress relaxation over a finite time scale, with the stress generation term corresponding to 2η(ϕ)D and the stress relaxation term corresponding to $\;\frac{-\tau }{\lambda }$. The full Oldroyd-B description would include the upper-convected derivative terms, such as ν·∇τ, −(∇ν)τ, $-\tau {\left(\nabla \nu \right)}^{T}$, but these contributions are neglected here because the NIL filling process is low-speed and locally dominated, and the simplified formulation is numerically more stable under coarse-grid explicit stepping.

To avoid discontinuous material jumps across the interface, the model uses a smooth phase indicator

(18) $$M(\phi )=\frac{1}{1+exp(10\phi )}$$

so that the effective viscosity becomes

(19) $$\eta \left(\phi \right)=\eta_{air}+(\eta_{polymer}-\eta_{air})M(\phi )$$

This ensures that viscoelastic stress exists predominantly in the resist region while the air region remains mechanically weak, without generating nonphysical oscillations at the diffuse interface. The velocity field is then updated from the competition between stress divergence and mold-driven pressure potential. Using a mold field m, a simple pressure-like driving term can be written as

(20) $$p=\left(m-\phi \right)k_{p}$$

and the explicit velocity update becomes

(21) $$\nu^{n+1}=\nu^{n}+\frac{\left(\nabla \cdot \tau \right)-\nabla p}{\eta \left(\phi \right)+\varepsilon }∆t$$

This relation reflects the central NIL mechanism used here: mold geometry and viscoelastic response together drive resist redistribution. Hence, the final imprint morphology is governed by both inherited topography and material rheology.

The interface evolution itself is written as

(22) $$\frac{\partial \phi }{\partial t}+\nu \cdot \nabla \phi =\gamma_{s}\nabla^{2}\phi$$

where the Laplacian term ($\nabla^{2}\phi$) is used as a regularized smoothing term associated with interface stabilization. After filling, UV curing is introduced through a Beer–Lambert-type attenuation model,

(23) $$\frac{dI}{dz}=-\alpha I$$

(24) $$I\left(z\right)=exp\left(-\alpha \frac{d(z)}{N_{z}}\right)$$

and the local crosslinking field is defined as

(25) C(x,y,z)=I(z)M(ϕ)

The cured phase is then represented by

(26) $$\phi_{cured}=\phi -\gamma C$$

which provides a compact way to encode morphology locking after exposure. Finally, the measurable topography is reconstructed as a resist height map H(x,y) by locating the highest resist layer at each lateral coordinate. This map becomes the NIL-stage process-conditioned prior passed into the learning module.

The importance of this stage is twofold. First, it closes the physical gap between template topography and final post-imprint morphology. Second, it produces a field H(x,y) that is directly relevant to defect risk because incomplete cavity filling, residual non-uniformity, and void-prone zones are all morphology-level precursors of later functional failure. Thus, NIL is not treated as a cosmetic final rendering stage; it is a major contributor to the risk landscape.

#### 2.2.5. Physics-Consistent Prior and Virtual SEM Generation

Collectively, the maps

(27) $$\left\{\hat{t}\left(x,y\right),D\left(x,y\right),D_{prox}\left(x,y\right),∆CD\left(x,y\right),H(x,y)\;\right\}$$

define a physics-consistent prior over the way layouts deform under manufacturing variability. Starting from the design layout, the integrated process simulation produces the corresponding AII pattern, which is then converted into a virtual SEM-like representation for downstream stochastic learning. In the present paper, the virtual SEM generation step is used as an intermediate image interface and is not further elaborated here [2].

### 2.3. Physics-Augmented CNN and Uncertainty Calibration

**Figure 3:**
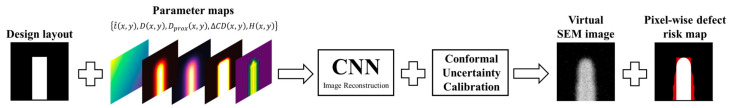
Stochastic risk prediction flow with physics-augmented CNN reconstruction and uncertainty calibration.

The stochastic prediction stage is formulated as a conditional image-to-image reconstruction problem in which the design layout and physics-consistent parameter maps are used to reconstruct the corresponding process-distorted SEM-like image, as shown in Figure 3. In this work, the term “physics-augmented” refers to the incorporation of process-derived physical parameter maps as structured multi-channel inputs to the CNN, rather than enforcing physical laws through loss function constraints as in conventional physics-augmented neural networks. Within this framework, the CNN does not operate on layout geometry alone. Instead, it is guided by stage-resolved physical descriptors, so that image reconstruction and risk inference remain linked to process-grounded priors rather than relying solely on statistical pattern matching. This design improves convergence, stability, and interpretability under realistic manufacturing variability.

#### 2.3.1. CNN Image Reconstruction

The learning task is formulated as a conditional mapping from the design layout and physics-consistent priors to the SEM-like target image:

(28) $$\hat{Y}=f_{\theta }\left(X,P\right)$$

where  X  denotes the design layout; $P=\left\{\hat{t},D,D_{prox},∆CD,H\right\}\;$ represents the physics-consistent parameter maps; $\;f_{\theta }$ is the CNN with trainable parameters  θ; and $\;\hat{Y}\;$ is the reconstructed SEM-like image.

**Figure 4:**
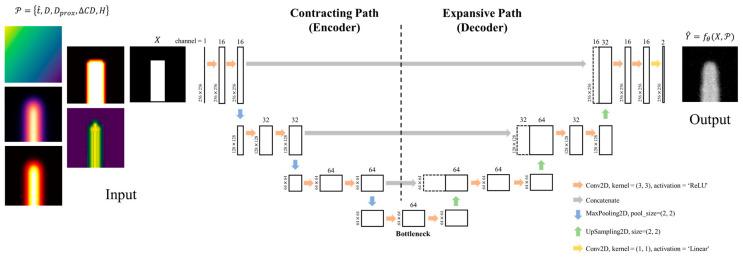
U-Net-based reconstruction framework from layout and physics priors to SEM-like output.

As illustrated in Figure 4, a U-Net-type architecture is adopted due to its capability to preserve multi-scale spatial information. The network architecture comprises stacked convolutional layers for feature extraction, downsampling (encoder) to obtain global context, and upsampling (decoder) to recover spatial resolutions. Skip connections are added to fuse low-level geometric features with high-level representations, enabling accurate reconstruction of edge-sensitive nanoscale structures.

To preserve both grayscale fidelity and edge sharpness, the model was trained using a hybrid loss function composed of an $\;L_{Unet}\;$ reconstruction term and a Sobel-edge consistency term:

(29) $$L_{hybrid}=L_{Unet}+\lambda_{e}L_{edge}$$

(30) $$L_{Unet}=\frac{1}{HW}\sum_{i,j}\left|{\hat{Y}}_{ij}-Y_{ij}\right|$$

(31) $$L_{edge}=mean\left(\left|\nabla_{Sobel}\hat{Y}-\nabla_{Sobel}Y\right|\right)$$

where $\nabla_{Sobel}\;$ denotes the discrete image gradient operator computed using Sobel filters, which approximate spatial derivatives along horizontal and vertical directions. This term enforces edge consistency between the predicted and ground-truth SEM images, thereby improving boundary fidelity and contour reconstruction accuracy. In the present implementation, the edge-weighting coefficient was fixed at $\;\lambda_{e}=0.3$.

This loss design is physically meaningful for NIL-related image reconstruction because the SEM target contains not only overall intensity information but also edge-sensitive contour features associated with linewidth definition, local corner rounding, and boundary distortion. Accordingly, the $\;L_{Unet}\;$ term preserves global appearance, whereas the Sobel-based term emphasizes structural consistency at pattern boundaries.

#### 2.3.2. Conformalized Quantile Regression for Pixel-Level Uncertainty

Because layout-to-SEM reconstruction is spatially heterogeneous and effectively stochastic, point prediction alone is insufficient. To quantify uncertainty, the model predicts lower and upper quantile bounds for each pixel. Let the predicted bounds be ${\hat{y}}_{ij}^{\left(q_{lower}\right)}\;$ and ${\hat{y}}_{ij}^{\left(q_{upper}\right)}$, where these denote the predicted lower and upper conditional quantiles at pixel (i,j), respectively. After conformal calibration, the prediction interval is given by

(32) $$I_{ij}=\left[{\hat{y}}_{ij}^{\left(q_{lower}\right)}-q^{*},{\hat{y}}_{ij}^{\left(q_{upper}\right)}+q^{*}\right]$$

where $\;q^{*}\;$ is the conformal correction obtained from the calibration set. The interval width then serves as a spatial descriptor of predictive uncertainty. In practical terms, narrow intervals indicate stable regions, whereas wider intervals indicate structurally ambiguous or process-sensitive regions. To further improve local reliability, the uncertainty-calibrated framework incorporates an encoder-frozen, outlier-weighted transfer fine-tuning strategy. The encoder parameters are fixed as

(33) $$\theta_{e}^{transfer}=\theta_{e}^{base},\;\;with\;\frac{\partial L}{\partial \theta_{e}^{transfer}}=0$$

so that the low-level representation learned from the larger dataset is preserved. Only the decoder and output layers are updated using pixel-level weights derived from conformal error maps. A weighted quantile loss can be written as

(34) $$L_{q}^{weighted}\left(y,\hat{y}\right)=\frac{1}{HW}\sum_{i,j}w_{ij}\left[max\left(q\left(y_{ij}-{\hat{y}}_{ij}\right),\left(1-q\right)\left(y_{ij}-{\hat{y}}_{ij}\right)\right)\right]$$

which directs corrective updates toward pixels that are under-covered or poorly reconstructed. This strategy is especially useful for edge-sensitive and hotspot-like regions, where global training alone may not allocate sufficient representational capacity. Previous NIL layout-to-SEM reconstruction results showed that this uncertainty-aware transfer strategy improved both pixel-level error and coverage performance, supporting its use here as the uncertainty-calibrated learning core.

#### 2.3.3. Pixel-Wise Risk Map Construction

After CNN-based SEM reconstruction, a pixel-wise attribution map was generated using Gradient-weighted Class Activation Mapping (Grad-CAM) to identify the spatial regions that most strongly influenced the model output. In this study, the attribution analysis was performed on the last convolutional feature layer selected for interpretation, and the final prediction score was defined as the spatial average of the reconstructed SEM response. Let $\;A_{ij}^{k}\in R^{H\times W}\;$ denote the k-th feature map in the selected convolutional layer. The gradient importance weight for channel k is computed as

(35) $$\alpha_{k}=\frac{1}{Z}\sum_{i}\sum_{j}\frac{\partial S}{\partial A_{ij}^{k}}$$

where  S  is the scalar prediction score and  Z  is the number of spatial locations in the feature map. This formulation corresponds to global average pooling of gradients over the feature map. The Grad-CAM map is then obtained by a weighted combination of feature maps:

(36) $$L_{Grad-CAM}=ReLU\left(\sum_{k}\alpha_{k}A^{k}\right)$$

The resulting map was normalized to the range [0,1] and resized to the original image resolution. It was then overlaid with the original mask image to visually depict the regions with the strongest contribution to the reconstructed SEM response.

In the context of this study, this Grad-CAM map serves as a pixel-wise vulnerability or response-attribution map. Regions with stronger activation indicate areas where the combined effect of layout geometry and physics-aware input channels exerts greater influence on the model prediction. Therefore, although the map is not a direct physical defect probability in the strict statistical sense, it provides an interpretable spatial indicator of where the model focuses when reconstructing process-distorted SEM appearance. This makes it useful as a practical pixel-wise risk-interpretation tool in the proposed virtual inspection framework.

### 2.4. Evaluation Metrics

To quantitatively evaluate the reconstruction accuracy, geometric fidelity, and uncertainty reliability of the proposed framework, two process-relevant metrics are defined in this study: the normalized CD error and the prediction interval coverage rate. These metrics are selected because the objective of the framework is not only to reconstruct SEM-like images, but also to assess whether the predicted pattern preserves manufacturing-relevant geometry and whether the associated uncertainty interval provides statistically meaningful reliability information.

The CD error, denoted as ∆CD, is used to quantify the geometric deviation between the target design layout and the simulated or reconstructed process output. In conventional lithographic metrology, the CD error is commonly defined as the absolute difference between the simulated or measured critical dimension and the target design critical dimension:

(37) $$\Delta CD=\left|{CD}_{sim}-{CD}_{design}\right|$$

where $\;{CD}_{design}\;$ is the target linewidth defined by the design layout, and $\;{CD}_{sim}\;$ is the linewidth obtained after the simulated process flow. In the present study, however, each pattern is represented as a discrete binary image. Therefore, the CD deviation is evaluated using an equivalent pixel-based definition. Specifically, because the target design and reconstructed output are represented as binary images, the pattern area is estimated by counting the number of pixels belonging to the patterned region. Therefore, the CD-related deviation is evaluated as the absolute difference in patterned-pixel counts between the target design and the simulated or reconstructed output:

(38) $$\Delta CD=\left|\sum_{x,y}1\left\{P\left(x,y\right)=0\right\}-\sum_{x,y}1\left\{\hat{P}\left(x,y\right)=0\right\}\right|$$

where  P(x,y)  denotes the target design pattern, $\hat{P}\left(x,y\right)\;$ denotes the simulated or reconstructed output pattern, and 1{·} is the indicator function. The value  P(x,y)=0  represents the patterned region in the binary image. Thus, this formulation directly quantifies the pixel-count difference between the intended and predicted patterned areas. To enable comparison across patterns with different areas, the CD error is further normalized by the target patterned area:

(39) $$Normalized\;\Delta CD=\frac{\left|\sum_{x,y}1\left\{P\left(x,y\right)=0\right\}-\sum_{x,y}1\left\{\hat{P}\left(x,y\right)=0\right\}\right|}{\sum_{x,y}\left\{P\left(x,y\right)=0\right\}}$$

From a process-physics perspective, ∆CD is a key indicator of geometric pattern fidelity. It directly reflects systematic linewidth deviation caused by proximity-induced overexposure or underexposure, nonlinear development response, etch-induced linewidth shrinkage, and imprint-related geometric distortion. Therefore, minimizing ∆CD is closely related to the objectives of optical proximity correction, process-window optimization, and defect-risk reduction. Unlike global image-level metrics, ∆CD focuses on geometry-level deviation and is therefore more directly connected to manufacturability. For example, two reconstructed images may exhibit similar global intensity or pixel-wise appearance but still differ in linewidth bias; in such cases, ∆CD provides a more sensitive indicator of process-relevant error. Conversely, localized boundary roughness may increase image-level error while producing only a limited change in overall CD. Therefore, CD error and image-level reconstruction metrics should be regarded as complementary rather than redundant.

To evaluate the reliability of the uncertainty estimation, the prediction interval coverage rate is further introduced. This metric quantifies whether the prediction interval produced by conformalized quantile regression successfully contains the target value at each pixel. Unlike deterministic reconstruction metrics, which measure the closeness between the predicted and target images, the coverage rate evaluates whether the model provides a statistically reliable uncertainty interval around its prediction. It is therefore a central metric for assessing the validity of the uncertainty-aware component of the framework.

Based on the calibrated prediction interval defined in Equation (32), the pixel-wise prediction interval coverage rate is used to quantify the fraction of target pixels that fall within the predicted uncertainty bounds. It is defined as

(40) $$Coverage=\frac{1}{HW}\sum_{i,j}1\left\{y_{ij}\in I_{ij}\right\}$$

where $\;I_{ij}\;$ is the calibrated prediction interval at pixel position  (i,j)  defined in Equation (32); H and W  are the image height and width; HW is the total number of pixels; $y_{ij}$ is the target pixel value; and 1{·} is the indicator function. A value of 1 is assigned when the target value lies within the calibrated prediction interval, and 0 otherwise. Therefore, the coverage rate represents the fraction of pixels whose true values are successfully covered by the predicted uncertainty interval.

A higher coverage rate indicates that the predicted interval more frequently contains the target value. However, coverage should not be interpreted independently of interval efficiency. A trivially wide interval may achieve high coverage but provides limited practical discrimination. Therefore, the purpose of conformalized quantile regression is not to maximize coverage unconditionally, but to construct prediction intervals that satisfy the target confidence level while remaining informative for spatial risk interpretation. In the present study, the nominal target coverage is set to 90%, corresponding to the lower and upper quantiles q = 0.05 and q = 0.95.

From the viewpoint of uncertainty quantification, coverage failure corresponds to interval miscoverage. Pixels that frequently fall outside the calibrated interval indicate locations where the model underestimates uncertainty or fails to reconstruct the local pattern response reliably. Such pixels often occur near pattern edges, high-curvature regions, dense pattern intersections, or process-sensitive areas where local geometry and process variation are strongly coupled. Therefore, the prediction interval coverage rate is not merely a statistical metric but also provides a basis for identifying spatially localized regions of elevated uncertainty and potential defect risk.

In this study, the same coverage criterion is applied when comparing the baseline model and the transfer-learning-enhanced model. This design ensures that improvements in coverage and outlier reduction are not caused by changing the evaluation threshold, but by the improved predictive capability and uncertainty calibration of the model itself. Accordingly, the combined use of normalized ΔCD and coverage rate enables the proposed framework to be evaluated from both the geometric-fidelity perspective and the uncertainty-reliability perspective. Together, these metrics provide quantitative support for assessing whether the model can reconstruct process-distorted patterns accurately while also producing statistically meaningful prediction intervals for pre-manufacturing risk analysis.

## 3. Results

### 3.1. End-to-End Demonstration of Physics-Grounded Pre-Manufacturing Virtual Inspection

This subsection demonstrates the end-to-end execution of the proposed framework on a representative layout, showing how a single input design is deterministically propagated through the integrated process twin and subsequently transformed into a stochastic, spatially resolved risk output. Starting from the NDA-safe binary layout, the physics-based stages generate intermediate inspection-equivalent patterns, namely ADI, AEI, and AII, that explicitly encode stage-by-stage pattern deformation. In parallel, the framework produces spatial parameter fields that capture the latent precursors of pattern failure, including thickness non-uniformity, proximity-modulated exposure non-uniformity, etch-induced linewidth loss, and imprint filling response. These maps serve two complementary roles: they provide physically interpretable descriptors of why certain regions become vulnerable, and they act as physics-consistent priors that constrain downstream learning and uncertainty-aware inference.

**Figure 5:**
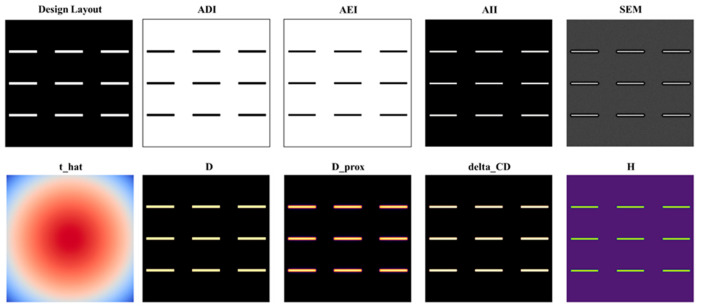
End-to-end physics-based virtual inspection and risk prediction pipeline.

The results show that the distortion observed in the final AII is not treated as a purely image-level artifact in Figure 5. Instead, it is explained through physically meaningful intermediate quantities. Regions exhibiting higher proximity accumulation in the effective dose field are associated with systematic CD modulation after development, while geometry-dependent etch effects further amplify linewidth erosion and microloading-like responses that vary across the same nominal pattern class. During imprint, the mold inherited from AEI imposes additional spatial constraints on resist redistribution, which yields a non-uniform topography and filling response. Importantly, because the framework retains spatial fields at each stage rather than reducing them to scalar process settings, it becomes possible to produce a continuous vulnerability representation: risk is predicted as a field that reflects local process sensitivity rather than as a post hoc binary defect decision.

Overall, this end-to-end result supports the central premise of the present study [2]: Pre-manufacturing virtual inspection becomes feasible when the process twin generates physically consistent intermediate patterns and parameter maps, which together enable spatially resolved risk estimation. This provides a direct pathway from layout geometry to actionable yield-risk insight while remaining both manufacturing-relevant and NDA-safe.

### 3.2. Quantitative Validation of Uncertainty Calibration and Computational Efficiency

**Figure 6:**
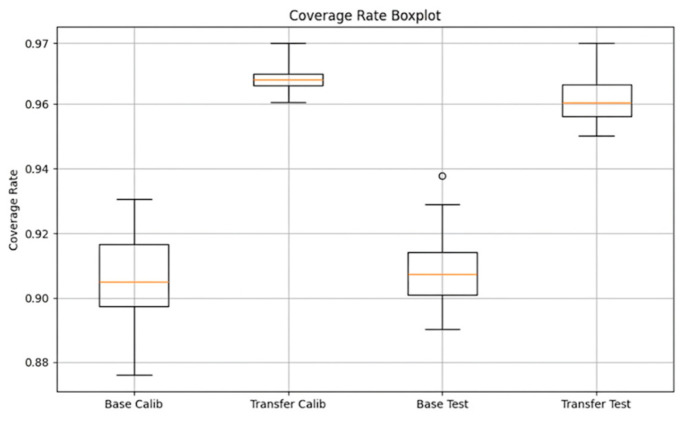
Coverage rate distribution for baseline and transfer models across calibration and test datasets.

To further substantiate the added value of the proposed CNN framework beyond physics-based simulation, quantitative evaluation was conducted on uncertainty calibration performance and computational efficiency using the calibration and test datasets. As shown in Figure 6 and Table 1, the baseline model yields coverage rates of 0.905 and 0.907 for the calibration and test datasets, respectively, which are close to the nominal 90% target but exhibit relatively larger variance. After applying outlier-weighted transfer learning with conformal calibration, the coverage rates increase to 0.964 and 0.960, while the corresponding standard deviations decrease significantly, indicating improved statistical stability and consistency of the prediction intervals.

**Figure 7:**
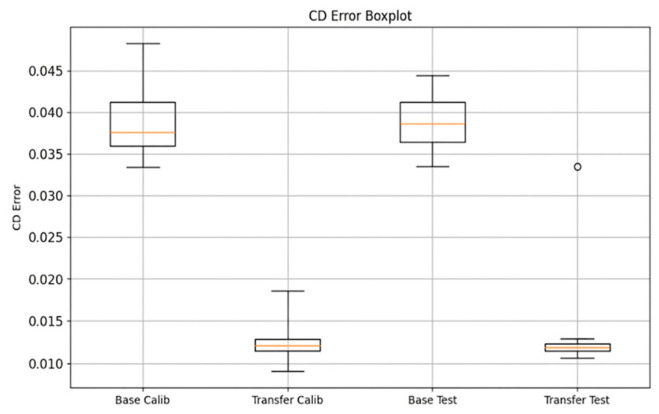
CD error distribution for baseline and transfer models across calibration and test datasets.

At the same time, the CD error is substantially reduced, as shown in Figure 7 and Table 1, from 0.0380 to 0.0125 in the calibration dataset and from 0.0390 to 0.0120 in the test dataset, demonstrating that the uncertainty-aware framework does not merely enlarge prediction intervals but instead achieves simultaneous improvement in both accuracy and reliability. This behavior is consistent with the theoretical objective of conformalized quantile regression, in which prediction intervals are adaptively calibrated to match the empirical error distribution while preserving coverage guarantees. Notably, the conformal calibration is performed using only 30 samples in the calibration set, yet the resulting coverage remains stable and close to the target confidence level, suggesting that the distribution-free nature of the method enables reliable uncertainty estimation even under limited data conditions.

**Figure 8:**
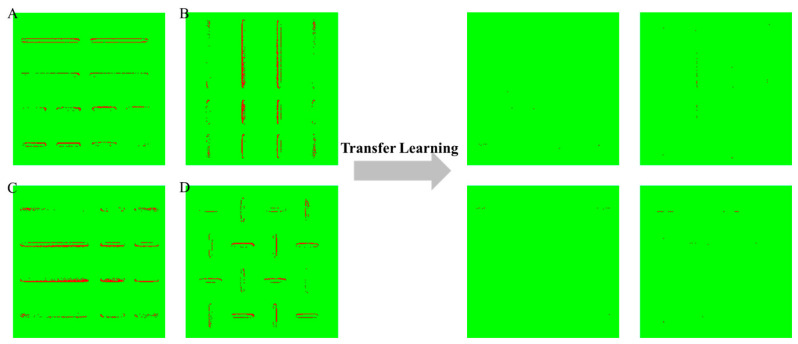
Outlier distribution before and after transfer learning. (**A**–**D**) Outlier distributions obtained from different design layouts.

From a spatial perspective, the outlier distribution maps shown in Figure 8 further illustrate the impact of uncertainty-aware transfer learning. Under the baseline model, prediction errors are primarily concentrated along pattern edges and high-curvature regions, reflecting local sensitivity to geometry-dependent process variations. After transfer learning optimization, these outliers are significantly reduced and become more spatially dispersed, indicating that the model has effectively corrected regions associated with high prediction uncertainty. Importantly, the same outlier-detection criterion is applied in both cases, ensuring that the observed improvement originates from enhanced model capability rather than evaluation bias.

In addition to predictive performance, the proposed framework also provides a substantial computational advantage compared to full physics-based simulation. The complete dataset consists of 250 layout samples, among which 195 samples are used for training and validation, 30 samples for conformal calibration, and 25 samples for testing. Training is performed on an NVIDIA RTX 3060 GPU, requiring approximately 36 min for the baseline model over 50 epochs and 10 min for transfer learning fine-tuning over 32 epochs with early stopping, corresponding to an average computational cost of approximately 43 s per epoch and 19 s per epoch, respectively. Once trained, the CNN performs inference on a single layout sample within sub-second time (on the order of 10^−1^ s), which is several orders of magnitude faster than the multi-stage physics-based simulation chain that involves iterative numerical solvers across coating, exposure, etching, and imprint processes. Therefore, the CNN serves as a high-speed surrogate model that preserves physics-consistent behavior while enabling rapid evaluation of layout-dependent defect risk.

Taken together, these results demonstrate that the proposed CNN framework provides quantifiable added value beyond direct simulation by improving prediction accuracy, stabilizing uncertainty estimation, and significantly accelerating inference. In this sense, the role of the CNN is not to replace the physical model, but to extend it into a computationally efficient and uncertainty-aware prediction tool that supports practical pre-manufacturing risk assessment.

### 3.3. Physical Consistency of Model Attribution via Grad-CAM Analysis

**Figure 9:**
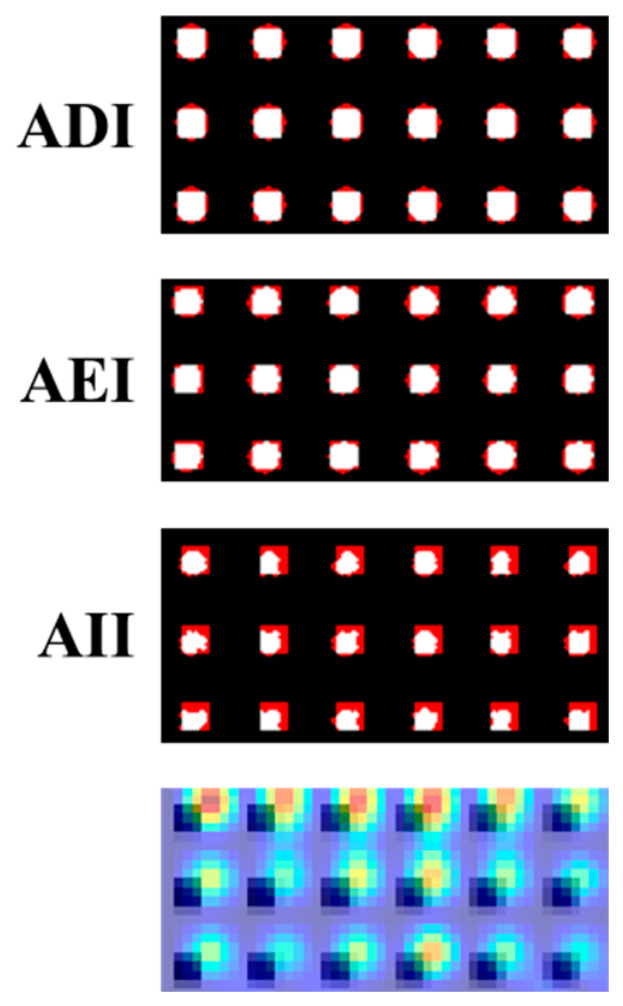
Error heatmaps of ADI, AEI, and AII relative to the design layout, and the corresponding pixel-wise risk map for a representative layout sample.

**Figure 10:**
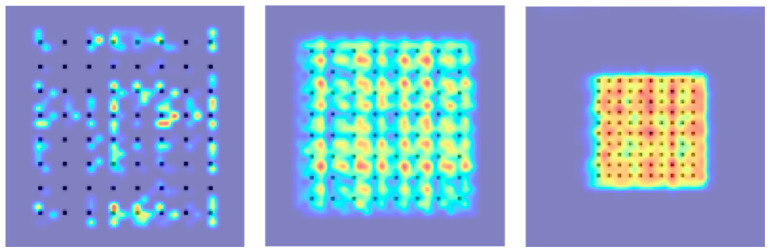
Risk-map comparison for patterns with identical contour extent but different local density.

To further examine whether the learned model attribution reflects physically meaningful process variations rather than numerical artifacts, a comparative analysis was conducted by relating the final pixel-wise risk map to the stage-wise error heatmaps obtained by comparing the design layout with the simulated ADI, AEI, and AII patterns. Figure 9 presents a representative dense-pattern sample, where the upper panels show the error distributions between the design layout and the intermediate process outputs ADI, AEI, and AII, while the lower panel shows the corresponding pixel-wise risk map generated from the Grad-CAM-based attribution analysis. Since the contour features in the full-layout view are relatively small and may be difficult to inspect visually, Figure 9 uses a magnified crop from the representative middle-density pattern shown in Figure 10 to more clearly present the spatial correspondence between stage-wise errors and the pixel-wise risk response.

It can be observed that the ADI, AEI, and AII error heatmaps all exhibit non-uniform spatial distributions, indicating that the deviation from the intended design is progressively accumulated and redistributed across the multi-stage process chain. In particular, several local regions showing stronger mismatch in the ADI, AEI, or AII stages are also reflected in the final pixel-wise risk map, suggesting that the model sensitivity is not arbitrarily distributed. Instead, it is related to process-sensitive locations where geometric distortion, local pattern deviation, or cumulative process perturbation be-comes more pronounced. This correspondence supports the view that the learned attribution retains meaningful links to the underlying process evolution.

At the same time, it should be emphasized that the core purpose of the pixel-wise risk map is not to directly predict the probability of defect occurrence. Rather, it is obtained through a gradient backpropagation mechanism that identifies the spatial regions to which CNN is most sensitive during SEM reconstruction. When a region exhibits a higher activation value in the Grad-CAM map, it indicates that the local geometry, physical parameter fields, or process-induced variation at that location exerts a stronger influence on the final model output. Such regions are typically also the locations in NIL where morphological deformation, local distortion, or defect generation is more likely to be amplified.

Therefore, although the ADI, AEI, and AII error distributions do not map one-to-one onto the final pixel-wise risk map, their partial spatial correspondence demonstrates that the attribution results are not merely numerical artifacts. In-stead, the pixel-wise risk map provides a physically interpretable indicator of process-sensitive regions, linking the CNN attention mechanism with stage-resolved process deviations. Minor differences remain expected because the Grad-CAM map reflects model sensitivity rather than direct geometric error itself. Even so, the overall agreement confirms that the integrated framework captures meaningful spatial vulnerability patterns and provides a practically useful basis for early defect-risk assessment.

### 3.4. Density-Dependent Risk Variation Under Identical Contour Extent

This subsection examines a key manufacturing-relevant behavior: even when the global contour extent of a pattern region is comparable, differences in local pattern density can induce markedly different risk distributions. Using representative cases with similar overall contour sizes, the results show that the predicted risk maps are not uniform across cases in Figure 10; instead, the risk reorganizes spatially and changes in magnitude as a function of density. This is an important distinction from binary inspection logic. Two patterns may occupy a similar area and appear comparable under coarse geometric descriptors, yet their process sensitivity and therefore defect propensity can differ substantially.

The observed density dependence is physically consistent with the coupled mechanisms embedded in the process twin. Firstly, the increased density results in increased interactions via proximity effects, leading to non-uniform dose accumulation and, hence, non-uniform effective exposure for a fixed nominal recipe. This may result in non-uniform bias and thinning during the development process, leading to vulnerable areas that may not be uniformly distributed across the array. Secondly, during the etching process, the increased density of the arrays increases the limitations in the transport process and microloading effects, leading to non-uniform variations in linewidth loss compared to relaxed or isolated arrays. Finally, during the imprint process, the increased density of the pattern environment increases the constraints on the flow pathways and may result in non-uniform residual layers, especially in the interior regions of the arrays.

A notable implication is that the same contour extent does not imply the same manufacturability. The risk maps provide a continuous indicator of this difference by revealing how density reshapes the spatial distribution of vulnerability. In practice, this enables pre-manufacturing screening of layouts that would otherwise appear equivalent under contour-based or rule-based criteria. Therefore, the density-dependent results support the need for pixel-wise risk prediction: the framework is sensitive to layout-induced process coupling, and it translates those couplings into interpretable, spatially resolved risk variations that can guide early-stage design and process decisions.

### 3.5. Why the Integrated Framework Is Stronger than Stage-Isolated or Purely Data-Driven Alternatives

The results above also clarify why an integrated framework is necessary. A stage-isolated model may reproduce one process step accurately, but it cannot reveal how upstream variation seeds downstream vulnerability. For example, a stand-alone imprint flow model would not indicate whether a filling anomaly originated from spin-coating thickness variation, exposure-induced CD modulation, or etch-driven density-dependent transfer loss. Conversely, a purely data-driven model may reconstruct SEM-like images effectively, but without process-conditioned priors, it becomes more difficult to attribute failure mechanisms or generalize under limited data. The present framework bridges this gap by preserving stage-resolved physical descriptors while using uncertainty-aware learning to infer stochastic defect risk.

The role of uncertainty calibration is especially important in this context. While the CNN predicts the SEM-like reconstruction, the calibrated prediction intervals indicate where the prediction is statistically less certain. The distinction is important because regions of significant physical distortion and regions of low prediction confidence are not necessarily coincident, although both are relevant for decision-making in manufacturing. The combination of reconstruction deviation and calibrated uncertainty results in a risk map that is spatially resolved and reliability-aware. In this sense, the framework functions not only as a predictive engine but also as a diagnostic tool for pre-manufacturing virtual inspection with quantified reliability.

A further advantage lies in its interpretability. The process twin retains a set of physically meaningful fields, while the learning stage transforms them into a pixel-wise risk distribution rather than collapsing them into a single decision label. This allows the user to inspect not only where risk is elevated, but also which physical precursor is most likely responsible. Such traceability is especially valuable in advanced NIL, where dense layouts, transfer-sensitive structures, and repeated template usage require process screening before fabrication begins. Overall, the integrated framework is stronger because it treats defect propensity as the outcome of coupled physical mechanisms and stochastic variability, rather than as an isolated image-based event.

## 4. Discussion

The present study demonstrates a route from design layout to pre-manufacturing risk prediction by explicitly coupling process physics and uncertainty-aware learning. From a manufacturing perspective, the main significance lies in shifting the role of inspection from reactive defect recognition to proactive risk screening. Rather than waiting for defects to appear on fabricated wafers, the proposed framework estimates where vulnerability is likely to emerge by propagating the design through physically meaningful process stages. This shift is especially relevant for NIL because template-related or layout-dependent variability can influence a large number of wafers before conventional inspection detects the problem.

Another important point is that the framework preserves physical causality in a stage-resolved manner. The spin-coating model contributes a local thickness prior; the exposure and development stage convert layout context into dose- and threshold-dependent contour variation; the etch stage introduces density-sensitive transfer loss; and the imprint stage maps inherited mold geometry into filling-dependent topography. These fields are not auxiliary outputs added for convenience. They are the physical variables through which local geometry is translated into local risk. Therefore, the model does not merely indicate that a region is high-risk; it also provides a structured explanation of why that region becomes vulnerable. This is one of the main strengths of combining a process twin with a data-driven risk predictor.

The density-dependent results further emphasize that manufacturability cannot be inferred from layout geometry alone. Even when contour extent appears comparable, the local pattern environment changes how exposure accumulates, how transport proceeds during etching, and how resist redistributes during imprinting. In dense layouts, these couplings are typically stronger and more spatially entangled. As a result, risk becomes more distributed and, in some cases, more interior-concentrated. This observation has practical implications for NIL-aware correction, template qualification, and layout screening, because apparently similar geometries may require different safety margins once process coupling is considered.

At the same time, several limitations should be recognized. First, the present framework is demonstrated on representative NDA-safe layouts rather than production masks, although this choice is deliberate to preserve reproducibility and proprietary safety. Second, the NIL stage is parameterized to capture the dominant rheological and interface-evolution mechanisms without fully resolving chemistry-specific curing kinetics or all mold–resist interfacial interactions. Third, the virtual inspection output is intended as a pre-manufacturing decision aid rather than a direct substitute for all in-fab metrology. While the proposed framework demonstrates the feasibility of integrating physics-based modeling with uncertainty-aware learning, the current study relies on simulation data without direct comparison to experimental SEM images due to data availability constraints. Therefore, the results should be interpreted as a proof-of-concept demonstration rather than a fully calibrated industrial solution. Future work will focus on (i) incorporating measured SEM datasets, (ii) calibrating process parameters with wafer-level metrology, and (iii) developing hybrid physics–data calibration workflows. Accordingly, broader deployment would still benefit from calibration against experimental ADI, AEI, AII, and SEM observations across a wider recipe space. Even so, the current results already show that the integrated framework provides a useful and physically interpretable basis for early yield-risk assessment.

Future work may extend the framework in several directions. One direction is recipe diversification, so that the same layout can be evaluated under different process windows and risk sensitivities. Another is modular transfer to related patterning, and pattern-transfer flows beyond NIL. A third is a tighter calibration against experimental SEM and inspection data, which would strengthen quantitative deployment while preserving the same stage-resolved architecture. In all cases, the central value of the present framework remains the same: it links layout geometry, process physics, and stochastic uncertainty into a unified pre-manufacturing inspection methodology.

## 5. Conclusions

This study presented an integrated physics-based and data-driven framework for defect prediction in advanced nanoimprint lithography toward inorganic semiconductor patterning. By linking an NDA-safe layout database, a physics-based process twin, and a physics-augmented CNN with conformal uncertainty calibration, the proposed system translates design layouts into spatially resolved defect-risk maps before fabrication. Unlike conventional post-fabrication inspection pipelines that rely on binary defect labels, the framework predicts defect vulnerability as a continuous field, capturing layout-dependent sensitivity arising from coupled process physics and stochastic variability.

Two conclusions are especially important. First, a pre-manufacturing virtual inspection can be implemented in an end-to-end and physically interpretable manner when the simulation chain preserves stage-resolved precursor fields from spin coating, exposure/development, etching, and imprinting. Second, patterns with comparable contour extent may still exhibit distinctly different risk distributions when local density changes, indicating that manufacturability is governed not only by nominal geometry but also by local pattern environment. These findings support the use of uncertainty-aware, physics-grounded virtual inspection for early yield-risk screening in advanced NIL.

Overall, the proposed framework provides a practical bridge between deterministic process understanding and stochastic defect-risk inference. It offers a physically interpretable basis for future density-aware correction, template qualification, and process optimization in NIL-based semiconductor manufacturing.

## Figures and Tables

**Table 1:** Quantitative comparison of CD error and coverage rate.

Dataset	Model	∆CD		Coverage	
		Mean	STD	Mean	STD
Calibration	Baseline	0.0380	0.0035	0.905	0.0110
	Transfer	0.0125	0.0020	0.964	0.0020
Test	Baseline	0.0390	0.0030	0.907	0.0090
	Transfer	0.0120	0.0015	0.960	0.0040

## Data Availability

The data used in this study are not publicly available due to institutional restrictions and industry confidentiality agreements.

## Abbreviations

The following abbreviations are used in this manuscript:

NIL	Nanoimprint Lithography
EBL	Electron-Beam Lithography
RIE	Reactive Ion Etching
NDA	Non-Disclosure Agreement
CNN	Convolutional Neural Network
UQ	Uncertainty Quantification
ARDE	Aspect-Ratio-Dependent Etching
CD	Critical Dimension
ADI	After Development Inspection
AEI	After Etching Inspection
AII	After Imprint Inspection
SEM	Scanning Electron Microscopy
Grad-CAM	Gradient-Weighted Class Activation Mapping

## Appendix A

### Appendix A.1. Spin-Coating Model Setting Parameters

**Table A1:** Setting parameters.

Parameter Name	Symbol	Value	Unit	Where Used
Wafer radius	R	50.0	mm	Equation (4): radial scaling
Reference spin speed	$\omega_{0}$	4000	rpm	Thickness scaling
Radial correction coefficient	$a_{2}$	−0.05	-	Equation (4)
Radial correction coefficient	$a_{4}$	−0.02	-	Equation (4)

### Appendix A.2. EBL Model Setting Parameters

**Table A2:** Setting parameters.

Parameter Name	Symbol	Value	Unit	Where Used
Nominal dose	D	120.0	µC/cm^2^	D(x,y)
Gaussian weight	$A_{1}$	0.74	-	Proximity kernel
Lorentzian weight	$A_{2}$	0.26	-	Proximity kernel
Lorentzian scale	γ	45	nm	Backscattering range
Threshold ratio	η	0.56	-	Development threshold
Thickness sensitivity	α	0.2	-	Equation (7)

### Appendix A.3. RIE Model Setting Parameters

**Table A3:** Setting parameters.

Parameter Name	Symbol	Value	Unit	Definition
Base vertical etch rate (Si)	$R_{base}$	2.8	nm/s	Equation (9)
Lateral-rate scaling (ion-assisted)	$r_{lat}$	0.03	-	Equation (9)
Chemical factor (Resist)	$f_{chem,R}$	0.06	-	Equation (9)
Chemical factor (Si)	$f_{chem,Si}$	0.12	-	Equation (9)
ARDE strength	α	4.5	-	$f_{acc}\left(AR\right)$
ARDE nonlinearity	p	2.5	-	
Lateral passivation factor	γ	2.6	-	$f_{lat}\left(AR\right)$
Lateral attenuation exponent	β	1.5	-	

### Appendix A.4. NIL Model Setting Parameters

**Table A4:** Setting parameters.

Parameter Name	Symbol	Value	Unit	Definition
Initial resist thickness	$h_{0}$	50	nm	Equation (15): initial filling layers
Vertical grid spacing	Δz	3	nm	Equation (15): layer discretization
Relaxation time	λ	0.2	s	Equation (16): viscoelastic relaxation
Polymer viscosity	$\eta_{polymer}$	2.0	Pa·s	Equation (19): resist viscosity
Air viscosity	$\eta_{air}$	0.1	Pa·s	Equation (19): background viscosity
Pressure scaling factor	$k_{p}$	1.0	-	Equation (20): mold-driven pressure
Numerical stabilizer	ε	1 × 10^−6^	-	Equation (21): avoid singularity
Surface smoothing coefficient	$\gamma_{s}$	0.3	-	Equation (22): interface regularization
Absorption coefficient	α	0.5	-	Equations (23) and (24): Beer–Lambert law
Curing coupling coefficient	γ	0.2	-	Equation (26): morphology locking
